# Ideal cardiovascular health status and its association with socioeconomic factors in Chinese adults in Shandong, China

**DOI:** 10.1186/s12889-016-3632-6

**Published:** 2016-09-07

**Authors:** J. Ren, X. L. Guo, Z. L. LU, J. Y. Zhang, J. L. Tang, X. Chen, C. C. Gao, C. X. Xu, A. Q. Xu

**Affiliations:** Shandong Provincial Center for Disease Control and Prevention, Preventive Medical Institute of Shandong University, Jinan, China

**Keywords:** Cardiovascular disease, Ideal cardiovascular health metrics, Socioeconomic status

## Abstract

**Background:**

Cardiovascular disease (CVD) is the leading cause of morbidity and mortality in the world. In 2010, a goal released by the American Heart Association (AHA) Committee focused on the primary reduction in cardiovascular risk.

**Methods:**

Data collected from 7683 men and 7667 women aged 18–69 years were analyzed. The distribution of ideal cardiovascular health metrics based on 7 cardiovascular disease risk factors or health behaviors in according to the definition of AHA was evaluated among the subjects. The association of the socioeconomic factors on the prevalence of meeting 5 or more ideal cardiovascular health metrics was estimated by logistic regression analysis, and a chi-square test for categorical variables and the general linear model (GLM) procedure for continuous variables were used to compare differences in prevalence and in means among genders.

**Results:**

Seven of 15350 participants (0.05 %) met all 7 cardiovascular health metrics. The women had a higher proportion of meeting 5 or more ideal health metrics compared with men (32.67 VS.14.27 %). The subjects with a higher education and income level had a higher proportion of meeting 5 or more ideal health metrics than the subjects with a lower education and income level. A comparison between subjects with meeting 5 or more ideal cardiovascular health metrics with subjects meeting 4 or fewer ideal cardiovascular health metrics reveals that adjusted odds ratio [OR, 95 % confidence intervals (95 % CI)] was 1.42 (0.95, 2.21) in men and 2.59 (1.74, 3.87) in women for higher education and income, respectively.

**Conclusions:**

The prevalence of meeting all 7 cardiovascular health metrics was low in the adult population. Women, young subjects, and those with higher levels of education or income tend to have a greater number of the ideal cardiovascular health metrics. Higher socioeconomic status was associated with an increasing prevalence of meeting 5 or more cardiovascular health metrics in women but not in men. It’s urgent to develop comprehensive population-based interventions to improve the cardiovascular risk factors in Shandong Province in China.

## Background

Cardiovascular disease (CVD) is the leading cause of morbidity and mortality in the world [1]. It’s still the most threatening disease influencing the health of the Chinese population and is expected to increase with aging of the population and further economic development [2]. Aging, hypertension, obesity, dyslipidemia and smoking have long been recognized as major risk factors of CVD in adults. The likelihood of developing CVD is significantly increased if one or more of these risk factors are present at the same time [3–8].

In 2010, a goal released by the American Heart Association (AHA) Committee focused on the primary reduction in cardiovascular risk [9]. The AHA defines the ideal cardiovascular health as the simultaneous presence of 4 ideal health behaviors (nonsmoking, body mass index (BMI) < 25 kg/m^2^, physical activity at goal level, and diet consistent with current guideline recommendations) and 3 ideal health factors (untreated blood pressure <120/80 mm Hg, untreated total cholesterol < 5.17 mmol/L (200 mg/dL) and untreated fasting glucose < 5.60 mmol/L (100 mg/dL)). Furthermore, it’s emphasized to monitor the race disparities in the use of the new impact goal. Recent studies have shown the low prevalence of the ideal cardiovascular health and its significant association with socioeconomic factors among adults [10–13]. However, studies concerning the ideal cardiovascular health in adult are still rare in china.

In this study, firstly, the status of ideal cardiovascular health metrics was assessed according to the definition of AHA in a Chinese population living in Shandong. Secondly, the socioeconomic factors in relation to the ideal cardiovascular health metrics in Chinese adults were investigated.

## Methods

### Study population

The Shandong province and the Chinese Ministry of Health (MOH) collaborative Action on Salt reduction and Hypertension (SMASH) program has been conducted in Shandong province from 2011 to 2015. A population-based cross-sectional base-line survey of SMASH was conducted in 2011, and a multi-stage (4-stage) cluster random sampling method was used to recruit a representative sample of the general population aged 18–69 years old and living in Shandong province for at least 5 years. Firstly, 20 districts/counties were selected after stratification by geographic distribution (Eastern, Central Southern, and North Western), and by residence status (urban and rural). Secondly, three townships (in rural areas) or two streets (in urban areas) were selected from each selected county or district by using a proportional probability sampling (PPS) method. Thirdly, also using PPS, three villages/communities were selected from each sampled township/street. Finally, about 50–60 households were selected by random sampling method in every selected village/community, and a total of 15600 individuals were invited to the survey,and the number of participants was 14230 (giving a response rate of 91 %). Those selected but did not attend the survey were replaced by adults with similar profiles from the same community or village, and a total of 1120 adults were replaced. The inclusion criteria for the current study were: individuals with no data missing for age, BMI, waist circumference (WC),smoking status, diet status, physical activity status, blood pressure measurements, lipids and fasting plasma glucose. A total of 15350 subjects with full required information were included.

### Data collection and biochemical variables determination

Participants were interviewed by a trained doctor or nurse. Information on dietary intake, smoking and physical activity was collected via a detailed questionnaire. Weight and height were tested with participants wearing light clothes and without shoes. BMI was calculated by dividing body weight (kg) by height squared (m^2^). Blood pressure was measured by a professional doctor with a standardized mercury sphygmomanometer (Yuyue, China) after at least 5 min rest. Three consecutive blood pressure readings, apart by at least 30 s, were taken from the right arm of seated subjects in a quiet room, and the average of the three readings was used in the data analysis. Fasting glucose levels (FPG) were determined by the glucose oxidase method and total cholesterol (TC) were determined by enzymatic method.

### Cardiovascular health metrics

The AHA definition of the ideal cardiovascular health metrics consists of 7 factors, and each factor was further classified into ideal, intermediate or poor categories. Participants who reported nonsmoking (never smoking or quitting >12 months) were categorized as having an ideal smoking status, participants who reported former smoking (quitting <12 months) were classified as having an intermediate smoking status, and those who reported current smoking (smoking daily regardless of the amount and type of smoking) were categorized as having a poor smoking status.

**Table 1:** Baseline characteristics of participants by gender

	Men	Women	Total
Number (%)	7683	7667	15350
Age (years)	41.2 (40.9,41.5)	41.5 (41.2,41.8)	41.4 (41.1,41.7)
School years > 9 (%)	27.1	19.3*	23.2
Personal income level(CNY/month)			
≤ 1000	85.14	84.91	85.03
1000–2000	12.20	13.35	12.76
≥ 2000	2.66	1.75	2.21
Current smoking (yes, %)	50.7	2.6*	26.6
Current drinking (yes, %)	67.6	11.3*	39.5
Diagnosed hypertension (%)	11.75	11.48	11.62
Body mass index (kg⁄m^2^)	24.4 (24.2,24.6)	24.5 (24.2,24.8)	24.5 (24.3,24.6)
Waist circumference (cm)	85.3 (84.6,85.9)	81.3 (80.6,81.9)*	83.3 (82.8,83.8)
Systolic blood pressure (mmHg)	124 (123,125)	118 (117,119)*	121 (120,122)
Diastolicblood pressure (mmHg)	80 (79,81)	77 (76,78)*	79 (78,80)
Fasting plasma glucose(mmol/L)	5.52 (5.45,5.59)	5.47 (5.40,5.53)	5.50 (5.45,5.55)
Total cholesterol (mmol/L)	4.36 (4.31,4.41)	4.37 (4.31,4.43)	4.37 (4.31,4.42)

Data are means (95 % confidence interval) or percentages indicated. *P < 0.05, men vs. women

Diet was evaluated by a food frequency questionnaire [14]. In defining health diet, we categorized the healthy diet into five components: 1) vegetables and fruits ≥500 g/day, 2) any fiber-rich whole grains in the diet (with legumes and cereals as the basic food), 3) fish ≥200 g/week [15], 4) sodium (<1500 mg/day) and 5) sugar-sweetened beverages (≤36 oz/week) in according to the recommended diet goals of AHA and the current Dietary Guidelines for Chinese Residents. Subjects with at least 4 of these 5 dietary components were categorized as having an ideal healthy diet score, subjects with 2 or 3 of these 5 dietary components were defined as having an intermediate diet score, and those with 0 or 1 component were classified as having a poor diet score.

The definition of physical activity includes the type and frequency of physical activity at work and during leisure time. Physical activity was divided into three levels: 1) Sedentary or light activity: taking no regular exercises, or relaxing walk after supper; 2) Moderate activity: jogging, walking briskly or dancing; 3) Vigorously active: intensive exercise, such as running, playing football or basketball. The International Physical Activity Questionnaire (IPAQ) was used to evaluate physical activity [16], which can be easily converted into minutes per week of moderate or vigorous exercise by IPAQ. The classification of AHA was used to categorize physical activity levels into ideal (≥150 min/week moderate intensity or ≥75 min/week of vigorous intensity), intermediate (1–149 min/week moderate intensity or 1–74 min/week vigorous intensity), and poor(sedentary or light activity) activity.

BMI was defined as ideal (<25 kg/m^2^), intermediate (25 to 29.9 kg/m^2^) or poor (≥30 kg/m^2^). Blood pressure was defined as ideal [systolic blood pressure (SBP)/diastolic blood pressure (DBP) < 120/80 mmHg, untreated], intermediate (SBP:120–139 or DBP:80–89 mmHg or treated to goal), or poor (SBP ≥ 140 mmHg or DBP ≥ 90 mmHg). TC was classified as ideal [<200 mg/dL (<5.26 mmol/L), untreated], intermediate [200 to 239 mg/dL (5.26–6.32 mmol/L) or treated to goal], or poor [≥240 mg/dL (≥6.32 mmol/L)]. FPG was classified as ideal [<100 mg/dL (<5.60 mmol/L), untreated], intermediate [100 to 125 mg/dL (5.60–6.90 mmol/L) or treated to goal], or poor [≥126 mg/dL (≥7.00 mmol/L)].

We also constructed an ideal cardiovascular health metrics score from zero to seven for each participant by recoding each component of the ideal cardiovascular health metrics as dichotomous variables in which 1 point was assigned for ideal status and 0 points for intermediate or poor status, and finally all subjects were assigned as having 0, 1, 2, 3, 4, 5, 6 or 7 points according to the number of ideal cardiovascular health metrics met at the ideal level.

### Determination of socioeconomic status (SES)

Socioeconomic status (SES) was evaluated in two dimensions: individual monthly income and education [17]. Personal monthly income was divided into three levels of ≤ 1000, 1000–2000, ≥ 2000 CNY (100 CNY = 16.3 USD). Education level was categorized into two levels (≤9 or > 9 school years).

### Statistical analysis

Data weight was standardized to the age and sex distribution for Chinese adults in 2010. Data were summarized as means (± standard error) for continuous variables and proportions for categorical variables of the participants, including the demographic characteristics and the distribution of the ideal cardiovascular health metrics. The ideal cardiovascular health metrics score was calculated. A chi-square test for categorical variables and the general linear model (GLM) procedure for continuous variables were used to compare differences in prevalence and in means among genders. The participants were divided into two groups according to the number of ideal cardiovascular health metrics. The subjects with 0, 1, 2, 3, and 4 ideal cardiovascular health metrics were combined as the reference group, because of the lack of subjects who met none of the ideal cardiovascular health metrics, and the participants with 5, 6, and 7 cardiovascular health metrics were combined into a single group. The socioeconomic factors were defined as independent variables. The association of the socioeconomic factors on the prevalence of meeting 5 or more cardiovascular health metrics was estimated by logistic regression analysis. Two models were evaluated. Firstly, the candidate risk factors, including age, school years, personal income level and socioeconomic status were fitted into a univariate logistic regression model one by one. Secondly, the risk factors listed above were entered into a multivariate logistic regression model at the same time. All data was analyzed with SAS 9.2 for Windows (SAS Institute Inc, Cary, NC). A *p*-value less than 0.05 (two tailed) was considered statistically significant.

## Results

The characteristics of the participants by gender were summarized in Table 1. The mean age was 41.2 years in men and 41.5 years in women. Compared with women, the men had higher education level, more obese and hypertensive. Current smoking and alcohol-drinking were more common in men than in women.

The definition and distribution of the ideal cardiovascular health metrics among men and women were summarized in Table 2. The prevalence of meeting ideal cardiovascular health metrics (such as meeting an ideal level of smoking, BMI, blood pressure, FPG and TC, respectively) is above 50 %, except for healthy diet score and physical activity, and the ideal categories of participants were 0.39 and 13.73 %, respectively. The fasting total cholesterol was the most prevalent ideal metrics (82.00 %), whereas the diet was the least prevalent (0.39 %). Compared with men, the women presented a higher prevalence of ideal health for current smoking status and blood pressure.

**Table 2:** Cardiovascular health metrics definition and their distribution (prevalence, %) among men and women

Health metrics		Definition	Men	Women	Total
Smoking	Ideal	Never or quit > 12 months	43.34	96.82*	70.75
	Intermediate	Former or quit <12 months	5.94	0.59	3.26
	Poor	Current	50.72	2.6	26.68
Body mass index	Ideal	<25 kg/m2	60.12	58.38	59.25
	Intermediate	25–29.9 kg/m2	32.31	32.33	32.32
	Poor	≥30 kg/m2	7.57	9.3	8.43
Diet	Ideal	4–5components	0.44	0.34	0.39
	Intermediate	2–3 components	99.53	99.63	99.58
	Poor	0–1 components	0.03	0.03	0.03
Physical activity	Ideal	≥150 min/week moderate intensity or 75 min/weekvigorous intensity or 150 min/week moderate + vigorous	13.78	13.68	13.73
	Intermediate	1–149 min/week moderate intensity or 1–74 min/week vigorous intensity or 1–149 min/wk moderate + vigorous	7.03	4.87	5.95
	Poor	None	79.19	81.45	80.32
Total cholesterol	Ideal	<5.17 mmol/L (200 mg/dl), without medcation	82.23	81.77	82.00
	Intermediate	5.17–6.20 mmol/L (200–239) mg/dl or treated to <5.17 mmol/L (200 mg/dl)	14.34	14.05	14.2
	Poor	≥6.21 mmol/L (240 mg/dl)	3.43	4.17	3.8
Blood pressure	Ideal	<120/<80 mm Hg, without medcation	43.41	62.24*	52.81
	Intermediate	SBP 120–139 or DBP 80–89 mm Hg or treated to goal	33.32	19.29	26.31
	Poor	SBP ≥ 140 or DBP ≥ 90 mm Hg	23.27	18.47	20.87
Fasting serum glucose	Ideal	<5.6 mmol/L (100 mg/dl)	59.23	63.33	61.28
	Intermediate	5.6–6.9 mmol/L (100–125 mg/dl) or treated to goal	33.91	31.14	32.52
	Poor	≥7.0 mmol/L (126 mg/dl)	6.87	5.53	6.20

*P < 0.05, men vs. women

As shown in Table 3, few participants (0.05 %) met all 7 cardiovascular health metrics. The prevalence of meeting 5 or more cardiovascular health metrics was 23.28 %, and the prevalence of meeting 1 or fewer cardiovascular health metrics was 7.15 %. Women, young subjects, and those with higher levels of education or income tended to have a greater number of the ideal cardiovascular health metrics. The women had a higher proportion of 5 or more ideal health metrics compared with men (32.67 VS.14.27 %), and the proportion of participants who had 5 or more ideal health metrics significantly decreased with age (41.34, 25.19, 16.46, 12.11, and 6.44 % in the subgroup s of 18 to 29, 30 to 39, 40 to 49, 50 to 59, and 60 to 69 years of age, respectively). The subjects with a higher education level had a higher proportion of 5 or more ideal health metrics than the subjects with a lower education level (38.51 VS.18.24 %), similarly, which was observed in the participants with a higher income (29.54 VS.22.63 %).

**Table 3:** Distribution (prevalence, %) of ideal cardiovascular health metrics in participants

Subjects group	Number	Number of ideal Cardiovascular Health Metrics							
		0	1	2	3	4	5	6	7
Total	15350	0.79	6.36	17.15	24.58	27.85	20.33	2.9	0.05
Age									
18-	3867	0.53	3.71	9.01	13.78	31.63	34.63	6.71	-
30-	3816	0.55	3.31	16.18	24.08	30.7	22.98	2.21	-
40-	3157	1.45	9.2	18.4	27.6	26.88	14.77	1.69	-
50-	2217	0.93	8.7	23.6	33.54	20.81	11.18	0.93	0.31
60-	2293	0.68	10.51	25.76	32.2	24.41	5.76	0.68	-
Sex									
Male	7683	1.56	8.69	21.13	26.72	27.63	12.53	1.74	-
Female	7667	.	3.92	12.99	22.35	28.08	28.46	4.11	0.1
Education level									
School years > 9	3596	0.39	2.92	13.23	17.12	27.82	29.18	9.14	0.19
School years < = 9	11754	0.92	7.44	18.39	26.94	27.86	17.53	0.92	-
Personal income level (CNY/month)									
≤ 1000	13051	0.77	6.21	17.2	25.18	28.01	20.09	2.54	-
1000–2000	1959	0.39	5.51	16.54	20.87	28.35	23.23	4.72	0.39
≥ 2000	340	2.27	2.27	18.18	15.91	31.82	20.45	9.09	-

The association of the socioeconomic factors on the prevalence of meeting 5 or more cardiovascular health metrics was presented in the Table 4. Multivariate logistic regression analysis revealed that the association of socioeconomic factors with the prevalence of meeting 5 or more cardiovascular health metrics differed in men and women. The comparison between subjects with meeting 5 or more ideal cardiovascular health metrics with subjects meeting 4 or fewer ideal cardiovascular health metrics revealed multivariate-adjusted odds ratio [OR, 95 % confidence intervals (95 % CI)] was 1.42 (0.95, 2.21) in men and 2.59 (1.74, 3.87) in women for higher education and income, respectively.

**Table 4:** Odds ratio (95 % confidence interval) of meeting 5 or more cardiovascular health metrics corresponding to a one standard deviation (SD) increase in continuous variables, and in relation to categorical variables as indicated

	Model 1	Model 2
Men (*n* = 7683)		
Age (year)	0.53 (0.44,0.65)	0.59 (0.48,0.74)
School years (>9 years VS < = 9 years)	1.01 (1.00,1.01)	0.73 (0.45,1.19)
Personal income level (CNY/month)		
≤ 1000	1.0	1.0
1000–2000	1.08 (0.64,1.82)	1.02 (0.69,1.49)
≥ 2000	2.02 (1.53,2.61)	1.46 (0.97,2.27)
Socioeconomic status		
Low income + low school year	1.0	1.0
Low income + high school year	0.61(0.35,1.02)	0.73 (0.42,1.28)
High income + low school year	0.78 (0.22,1.49)	0.84 (0.45,1.52)
High income + high school year	2.22 (1.47,3.36)	1.42 (0.95,2.21)
Women (*n* = 7667)		
Age (year)	0.34 (0.29,0.41)	0.42 (0.35,0.51)
School years (>9 years VS < = 9 years)	1.01 (1.00,1.01)	0.96 (0.72,1.29)
Personal income level (CNY/month)		
≤ 1000	1.0	1.0
1000–2000	1.51 (0.93,2.02)	1.04 (0.71,1.41)
≥ 2000	1.29 (0.84,2.04)	1.46 (0.95,2.23)
Socioeconomic status		
Low income + low school year	1.0	1.0
Low income + high school year	0.64 (0.24,1.65)	0.85 (0.63,1.10)
High income + low school year	1.25 (0.75,2.07)	1.19 (0.68,2.13)
High income + high school year	3.14 (2.10,4.70)	2.59 (1.74,3.87)
Total (*n* = 15350)		
Age (year)	0.44 (0.39,0.50)	0.51 (0.44,0.58)
School years (>9 years VS < = 9 years)	1.01 (1.00,1.01)	0.91 (0.62,1.23)
Personal income level (CNY/month)		
≤ 1000	1.0	1.0
1000–2000	1.34 (0.86,1.98)	1.03 (0.70,1.47)
≥ 2000	1.48 (0.97,2.27)	1.47 (0.96,2.25)
Socioeconomic status^a^		
Low income + low school year	1.0	1.0
Low income + high school year	0.63 (0.30,1.10)	0..83 (0.60,1.15)
High income + low school year	1.05 (0.69,1.63)	1.02 (0.59,1.75)
High income + high school year	2.70 (1.78,3.90)	1.85 (1.40,2.45)

^a^Socioeconomic status was defined according to both personal incomes (>1000 CNY vs. <=1000 CNY per person per month) and school years (>9 years vs. <= 9 years), was fitted in a separate model to replace personal income and school year. Model 1: The risk factors listed in the table were entered in to a univariate logistic regression model one by one; Model 2: The risk factors listed in the table were entered into a multivariate logistic regression model at the same time

## Discussion

In this population-based cross-sectional study, we found that the prevalence of meeting all 7 cardiovascular health metrics was very low. Our finding also indicated that higher socioeconomic status (higher education and income) was independently associated with an increased prevalence of meeting 5 or more cardiovascular health metrics in women but not in men.

Numerous studies have shown that the prevalence of ideal cardiovascular health is extremely low. Few participants (<2 %) met all 7 cardiovascular health metrics in the National Health and Nutrition Examination Survey including 44959 US adults (≥20 years) (NHANES) [13]. Another community-based population study including 1933 revealed that only one participant (0.1 %) met all 7 components of the AHA’s definition of ideal cardiovascular health, and less than 10 % of participants met 5 components of ideal cardiovascular health in all subgroups [18]. Our results add to previous findings that have demonstrated a low prevalence of healthy factors and lifestyles in the general population in Shandong Province. Moreover, some previous studies have shown that there is a significant association between the low prevalence of ideal cardiovascular health metrics and incidence of CVD among adults, and meeting a greater number of ideal cardiovascular health metrics is associated with a significantly low risk for CVD [13, 19]. With the low prevalence of healthy factors and lifestyles, it can be expected that CVD will continue to increase in the near future if there is no intervention [20, 21], so it’s urgently required to develop comprehensive population-based interventions to achieve greater number of ideal cardiovascular health metrics.

In the current study, only about 13.73 % of participants had an ideal physical activity, which is significantly lower than the proportion in American [13, 18], and about 80 % of participants had a sedentary or light physical activity in the current study. Along with the rapid development of internet, the increase in time spent on internet possibly contributed to reduced outdoor activities. Furthermore, lots of middle-aged or elderly people in Shandong Province in China prefer to a relaxing walk after dinner, and these participants were defined as “light active” in this study, which may partly explain why prevalence of the sedentary or light physical activity is so high in the current study. We also found the prevalence of an ideal healthy diet score was only 0.39 % in our study, which was the least prevalence of ideal cardiovascular health component, perhaps partly because there have been significant changes in Chinese diet habit during the past 20 years, such as decreased whole grains but increased animal product. In addition, previous reports from Shandong, China showed that the total standardized person daily dietary sodium intake estimated by 3-day dietary recall was 6090.1 mg, which was much higher than the recommended diet goals of AHA and might partly explain why the prevalence of an ideal healthy diet score was very low in our current study [22].

However, the socioeconomic status played a different role in men and women. Higher income and higher education were associated with an improved prevalence of meeting 5 or more cardiovascular health metrics in women but not in men, which consisted with a study conducted in American [23]. And some previous studies have also shown that lower educational attainment is associated with a higher risk for CVD [24–26]. This may be attributed to the fact that the women with higher education and income pay more attention to a healthy diet, weight reduction and physical activity. Moreover, higher education may facilitate the acquisition of positive social, psychological, economic skills and assets, and may provide insulation from adverse influences [24].

The women had a higher proportion of 5 or more ideal health metrics compared with men (32.67 VS.14.27 %) in our study, and this consist with previous report in US adults [20], which may be attributed to a higher prevalence of ideal health for current smoking status and blood pressure in women than in men. It might be because of greater self-care in women.

The study has several strengths. First, this is a population-based study which is a representative random sample of the general population in Shandong province. Large sample size provided high statistical power for data analyses. Second, the information regarding demography, economy, life style and anthropometry was available and supply the influent database to investigate. However, limitation should be considered. Since the current study was cross-sectional, which limited the ability to assess causal relationship.

## Conclusions

In summary, the prevalence of meeting all 7 cardiovascular health metrics was low in the adult population in Shandong Province. High socioeconomic status appeared to be associated with an improved prevalence of meeting 5 or more cardiovascular health metrics in women but not in men. Considering the high prevalence of obesity, physical inactivity and unhealthy diet, it’s urgent to develop comprehensive population-based interventions to improve the cardiovascular risk factors status in Shandong Province in China.

## Abbreviations

AHA: The American Heart Association
BMI: Body mass index
CI: Confidence interval
CVD: Cardiovascular disease
DBP: Diastolic blood pressure
OR: Odds ratio
SBP: Systolic blood pressure
SE: Standard error
TC: Total cholesterol
WC: Waist circumference
